# Machine learning identification of a novel vasculogenic mimicry-related signature and FOXM1’s role in promoting vasculogenic mimicry in clear cell renal cell carcinoma

**DOI:** 10.1016/j.tranon.2025.102312

**Published:** 2025-02-03

**Authors:** Chao Xu, Sujing Zhang, Jingwei Lv, Yilong Cao, Yao Chen, Hao Sun, Shengtao Dai, Bowei Zhang, Meng Zhu, Yuepeng Liu, Junfei Gu

**Affiliations:** a Department of Urology, The Second Hospital of Hebei Medical University, 215 Heping West Road, Shijiazhuang 050000, PR China; b Department of Nuclear Medicine, The Second Hospital of Hebei Medical University, 215 Heping West Road, Shijiazhuang 050000, PR China; c Hebei Medical University,361 Zhongshan East Road, Shijiazhuang, Hebei 050017, PR China

**Keywords:** Clear cell renal cell carcinoma;Vasculogenic mimicry;Machine learning;FOXM1;Tumor microenvironment;Prognosis;Therapeutic targets

## Abstract

**Background:**

Clear Cell Renal Cell Carcinoma (ccRCC), the predominant subtype of renal cell carcinoma (RCC), ranks among the most common malignancies worldwide. Vasculogenic mimicry (VM) plays a pivotal role in tumor progression, being closely linked with heightened chemoresistance and adverse prognosis in cancer patients. Nonetheless, the broader impact of vasculogenic mimicry-related genes (VRGs) on ccRCC patient prognosis, tumor microenvironment characteristics, and treatment response remains incompletely understood.

**Methods:**

Consensus clustering identified VRG-associated subtypes. We developed a machine learning framework integrating 12 algorithms to establish a consistent VM-related signature (VRG_score). The predictive value of VRG_score for ccRCC prognosis and treatment response was assessed. FOXM1′s clinical relevance was explored using the UCLCAN database. FOXM1 expression in tumor and adjacent tissues was assessed using Western Blotting, IHC, RNA-seq, and Chip-qPCR methods, and its regulatory mechanism was confirmed.

**Results:**

We examined VRG mutation and expression patterns in ccRCC at the gene level, identifying two distinct molecular clusters. A consensus VRG_score was formulated using a machine learning computational framework and Cox regression, displaying strong predictive power for prognosis and clinical translation. Additionally, FOXM1 was found to be upregulated in ccRCC, correlating with clinical pathological features and positively regulating PYCR1, thereby activating the PI3K/AKT/mTOR signaling pathway and promoting VM formation.

**Conclusion:**

This study constructed a VM-related signature and revealed that FOXM1 promotes VM formation in renal cell carcinoma through the PYCR1-PI3K/AKT/mTOR signaling axis, serving as a prognostic indicator and potential therapeutic target.

## Highlights

• Developed a robust Vasculogenic Mimicry-related signature (VRG_score) using a machine learning framework, demonstrating strong predictive power for both ccRCC prognosis and treatment response.
• Identified FOXM1 upregulation in ccRCC, which correlates with clinical features, positively regulates PYCR1, and promotes Vasculogenic Mimicry formation through the PYCR1-PI3K/AKT/mTOR-VEGFA signaling axis.
• Established VRG_score and FOXM1 as potential prognostic indicators and therapeutic targets for ccRCC.

## Graphical abstract

**Figure:**
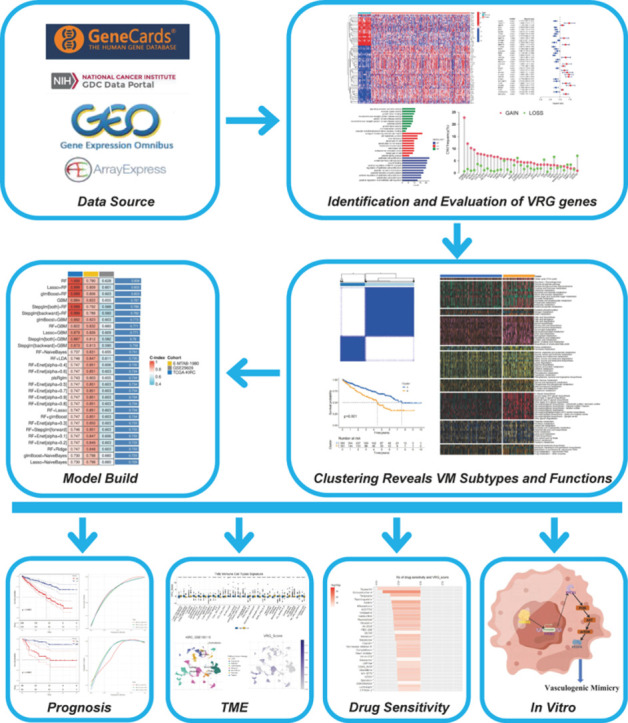

## Introduction

RCC is a typical primary malignant tumor. Despite significant advances in its treatment strategies over the years, RCC remains a widespread and challenging type of cancer [1]. ccRCC accounts for approximately 70 %−80 % of RCC cases and represents the most common pathological subtype of RCC [2]. Currently, the treatment of ccRCC primarily relies on the combined use of surgery and targeted drugs [3]. However, challenges in early diagnosis, drug resistance, and improvement of patient survival rates remain significant obstacles in the treatment of ccRCC [3,4]. Therefore, the search for new potential biomarkers for prognostic prediction and personalized therapy in ccRCC has become a hot topic in current cancer research.

Previous views suggested that endothelial cell-dependent tumor angiogenesis was the sole mode of blood supply [5]. However, as a highly vascularized solid tumor, the clinical efficacy of anti-angiogenic therapy in patients with clear cell renal cell carcinoma (ccRCC) remains unsatisfactory. Existing anti-angiogenic strategies primarily focus on the vascular endothelial growth factor (VEGF) or VEGF receptor (VEGFR) signaling pathways; however, despite some progress, the benefits derived from these approaches are often temporary and frequently fail to achieve long-term clinical responses. Vasculogenic mimicry, a distinct model of tumor microcirculation that does not rely on epithelial cells, is capable of transporting sufficient blood and nutrients to support tumor growth [6]. It has been reported that the formation of VM is closely associated with poor prognosis in various aggressive malignant tumors, including renal cell carcinoma [7], colorectal cancer [8], breast cancer [9], glioblastoma [10], and prostate cancer [11]. Furthermore, angiogenesis and VM often coexist in aggressive tumors, and anti-angiogenic drugs have minimal effects on VM [12]. Recent research indicates that tyrosine kinase inhibitors (TKIs), including sunitinib, may contribute to the development of VM [7]. This underscores how cancers are effectively stimulated to actively seek out nutrient supply despite the restriction of angiogenesis. As research continues to deepen, increasing attention is being paid to the potential of VM in cancer treatment, underscoring the necessity for combination treatment plans utilizing both anti-angiogenic and anti-VM agents.

In this study, VRGs were investigated using databases including The Cancer Genome Atlas (TCGA) and Geostationary Orbit (GEO). VRGs were selected through Cox regression, and based on this, consensus clusters were integrated. A machine learning-driven signature was constructed using 12 machine learning algorithms. In both the training and validation cohorts, the VRG_score demonstrated excellent prognostic value and robust performance in predicting responses to immunotherapy and drug-based interventions. We validated that VM formation is promoted by FOXM1-mediated PYCR1 through the PI3K/AKT/mTOR/VEGFA pathway. This study highlights the functional role of VRG features and reveals potential prognostic biomarkers for ccRCC, providing new insights into potential therapeutic targets and strategies for treating ccRCC.

## Materials and methods

### Data collection

From the TCGA database(https://portal.gdc.cancer.gov/), RNA expression data, somatic mutation data, Copy Number Variation(CNV) files, and related clinicopathological information of the TCGA-KIRC cohort. Subsequently, clinical parameters and normalized gene expression data were acquired by retrieving the GSE29609 [13] from the GEO database (https://www.ncbi.nlm.nih.gov/geo/) and the E-MTAB-1980 cohort [14] from the EMBL-EBI database (http://www.ebi.ac.uk/). To conduct analyses similar to those of E-MTAB-1980 and GSE29609, FPKM values of the TCGA-KIRC cohort were converted to TPM. We retrieved normalized gene expression data for 91 renal cancer patients from the ICGC-RECA-EU dataset available in the International Cancer Genome Consortium(ICGC) database (https://dcc.icgc.org/), of which 80 patients had complete clinical data. Following this, We accessed the GeneCards database(https://www.genecards.org/) and conducted a search using the keyword “Vasculogenic Mimicry” to identify all genes related to vascular processes. We filtered for genes with a relevance score > 1.0 from GeneCards and further excluded those genes with weaker associations to vasculogenic mimicry or lacking clear functional descriptions. Ultimately, we confirmed a set of 158 genes that are strongly associated with vasculogenic mimicry for subsequent analysis and research.

### Integrated multi-omics analysis of differential expression VRGs

The 'limma' package was utilized to identify differentially expressed VRGs between tumor-adjacent tissues and tumor tissues. Significance criteria were set at FDR 〈 0.05 and |logFC| 〉 1. The differential genes were subjected to Gene Ontology(GO) analyses, as well as Kyoto Encyclopedia of Genes and Genomes(KEGG) pathway analysis using the 'ClusterProfiler' package [15]. GeneMANIA(https://genemania.org/) was utilized to visualize the close interactions among VRGs [16]. The 'MAFTOOLS' package was utilized to describe the somatic mutations of these genes in patients [17]. The 'GISTIC2.0′ package was employed to characterize the CNV status, chromosomal information, and gain or loss status of each gene [18].

### Identification of different VM patterns based on unsupervised clustering

Following the merger of TCGA-KIRC and GSE29609 datasets, batch effects were rectified using the 'SVA' R package. The ConensusClusterPlus software package was employed for unsupervised clustering, iterating 1000 times to ensure the stability of these molecular subtypes. Principal Component Analysis (PCA) was performed to assess and compare the significant differences in transcription levels across various clusters. Additionally, three-dimensional Principal Component Analysis (3D PCA), Uniform Manifold Approximation and Projection (UMAP), and t-Distributed Stochastic Neighbor Embedding (t-SNE) were employed to further visualize and confirm the distinctions among the clusters. The transcriptional regulatory network was constructed using the 'RTN' package [19]. Kaplan-Meier (KM) survival plots were utilized to assess the overall survival (OS) rate and progression-free survival(PFS) rate of different clusters. Gene set variation analysis (GSVA) was performed using the KEGG gene set to identify biological functional differences of VRGs. Lastly, we compiled metabolism-related pathways from KEGG and calculated the correlation between VM subtypes and specific biological processes using the Single-sample gene set enrichment analysis (ssGSEA) method.

### Construction and clinical relevance assessment of VRG_Score

We selected the TCGA-KIRC cohort as the training set, and E-MTAB-1980 and GSE29609 as validation sets. To construct a robust prognostic model, we applied twelve diverse machine learning algorithms, including least absolute shrinkage and selection operator (Lasso), Ridge, Stepwise Generalized Linear Model (Stepglm), extreme gradient boosting machine (XGBoost), Random Forest (RF), Elastic Net (Enet), Partial Least Squares Regression for Generalized Linear Models (plsRglm), Generalized Boosted Regression Modeling (GBM), Naive Bayes, Linear Discriminant Analysis (LDA), Generalized Linear Model Boosting (glmBoost), and Support Vector Machine (SVM). Employing a tenfold cross-validation framework, using survival/death as the clinical outcome, fitted the diagnostic model based on the training dataset (TCGA-KIRC). For each algorithmic combination, we computed the area under the receiver operating characteristic curve (AUC) across all validation datasets. The model that yielded the highest mean AUC, reflecting optimal predictive performance, was selected as the optimal diagnostic model. Through a systematic arrangement of these algorithms, we generated 113 unique combinations to identify consistent regulatory genes that demonstrate strong accuracy and stability. A signature capable of predicting the survival of ccRCC patients, termed VRG_score, was established through Cox regression. KM and receiver operating characteristic (ROC) curve analyses were performed to assess the predictive performance of the VRG_score. The potential of the VRG_Score as an independent prognostic factor was subsequently assessed using multivariate and univariate Cox regression models. Classification analysis was performed to investigate whether the VRG_Score maintained predictive reliability across various subgroups based on multiple clinical factors. The establishment of a predictive nomogram provided valuable clinical prognostic standards for risk scoring and other clinical pathological features of ccRCC patients, particularly for prognosis at 1, 3, and 5 years. To validate the clinical reliability of the developed nomogram, calibration curve analysis and Decision curve analysis (DCA) were conducted subsequently.

### Molecular and immunological characteristics of VRG_Score

Signals associated with immune infiltration, Tumor microenvironment (TME) cell types, and response to immune treatment were investigated using the ‘IOBR’ package [20]. Scores for each sample were computed and normalized, facilitating a comprehensive evaluation of immunological differences between high and low VRG_score. Subsequently, a comparison was conducted between the Tumor neoantigen (TNB) and Tumor mutational burden (TMB) of the two patient groups. We retrieved the GSE159115 dataset from the TISCH2 (http://tisch.comp-genomics.org) database and systematically examined the heterogeneity of VRG_score across different cell types [21].

### Drug sensitivity assessment

Information on drug sensitivity was gathered from CTRP [22], PRISM [23], and GDSC [24]. As a gauge of drug sensitivity, AUC data are available for both CTRP and PRISM, whereas IC50 values are available for GDSC. AUC values that are lower suggest that a chemical is more sensitive to therapy. The possible reaction of ccRCC patients to particular medications is shown by the correlation coefficients between VRG_score and Half maximal inhibitory concentration (IC50) (or AUC).

### In vitro cell culture and maintenance

The HK2, 786-O, 769P, OSRC2, and A498 cell lines were obtained from the American Type Culture Collection (ATCC, Manassas, VA). All cells were authenticated through short tandem repeat (STR) DNA profiling and tested yearly for mycoplasma contamination using the Universal Mycoplasma Detection Kit (ATCC). The cell lines were cultured in Dulbecco's Modified Eagle's Medium (DMEM, Invitrogen, Grand Island, NY) supplemented with penicillin (25 units/ml), 10 % fetal bovine serum (FBS), streptomycin (25 μg/ml), and 1 % l-glutamine. All cultures were maintained in a humidified incubator at 37 °C with 5 % (v/v) CO_2_.

### Cell transfection

The expression of FOXM1 and PYCR1 was modulated using siRNA targeting FOXM1, siRNA targeting PYCR1, and a PYCR1 overexpression plasmid (GenePharma, Suzhou, China), respectively. Before proceeding with the subsequent experiment, the 786-O and 769P cell lines were incubated with siRNA-lipid complexes for 8 h. Subsequently, they were replenished with fresh culture medium and allowed to incubate for an additional 48 h. The supplementary Table provides details of the sequences used.

### Detection of mRNA expression by RT-qPCR

The Total RNA Extraction Kit (SEVEN, Beijing, China) was used for RNA extraction.1 μg of RNA was reverse transcribed using the reverse transcription kit (Yeasen, Shanghai, China). RT-qPCR was conducted to determine the expression levels utilizing the Bio-Rad CFX96 system with SYBR green. The 2−^ΔΔ^CT method was used to normalize the CT values. Supplementary Table provides detailed information on the primers used.

### Western blotting assay

RIPA whole-cell lysis buffer was utilized to lyse the cells. Proteins were separated by 8–12 % SDS-PAGE electrophoresis and then transferred to PVDF membranes (Merck, Darmstadt, Germany) using a semi-dry transfer method. The PVDF membranes were then blocked in a solution of 5 % skim milk in TBST for 2 h, followed by three washes with TBST(5 mins each) and overnight incubation at 4 °C. The following day, after washing the membranes three times with TBST(5 mins each), they were then subjected to a two-hour incubation at room temperature with secondary antibodies targeting either rabbit or mouse. Subsequently, another round of washing was carried out, following the same protocol of three washes with TBST(5 mins each). Bands were visualized using an ECL chemiluminescence detection system. Supplementary Table provides detailed information on the Antibodies used.

### Immunohistochemical stainings and evaluation

The Immunohistochemistry (IHC) staining was performed according to a previously established protocol [25]. Paraffin-embedded renal cell carcinoma tissue sections were treated with anti-FOXM1 antibody. The specimens were examined using a microscope(Leica DM2500P, Germany).

### Chromatin immunoprecipitation assay (ChIP)

The ChIP experiment was conducted following a previously established protocol [26]. After cross-linking with formaldehyde, the cell lysates were sonicated, followed by overnight incubation with IgG and anti-FOXM1 antibody. The antibody/protein/DNA complexes were collected the next day according to the experimental protocol. Target sequences within the PYCR1 gene promoter were designed, and the results were validated using RT-qPCR.

### Matrigel tube formation assay

The Matrigel matrix (Corning, Corning, NY) was thawed and gently mixed until homogeneous using a cooled pipette. A 96-well plate was then filled with 50 μl of the matrix solution in each well. The plate was incubated for sixty minutes at 37 °C to allow the matrix solution to solidify. Tumor cells (2 × 10^^5^) that had been treated were added to the wells containing the solidified Matrigel matrix. The 96-well plate was then incubated for >6 h in the cell culture incubation system. The same experiment was repeated three times. The number of formed tubes was measured using AngioTool software.

### Statistical analysis

Statistical data analysis and graphical representation were conducted using R (version 4.13) and GraphPad Prism 8.0. Pearson or Spearman coefficients were employed to assess the correlation between variables. Statistical significance was considered when the p-value for each statistical computation was <0.05.

## Results

### Multi-omics landscape of VRGs in ccRCC

**Fig. 1:**
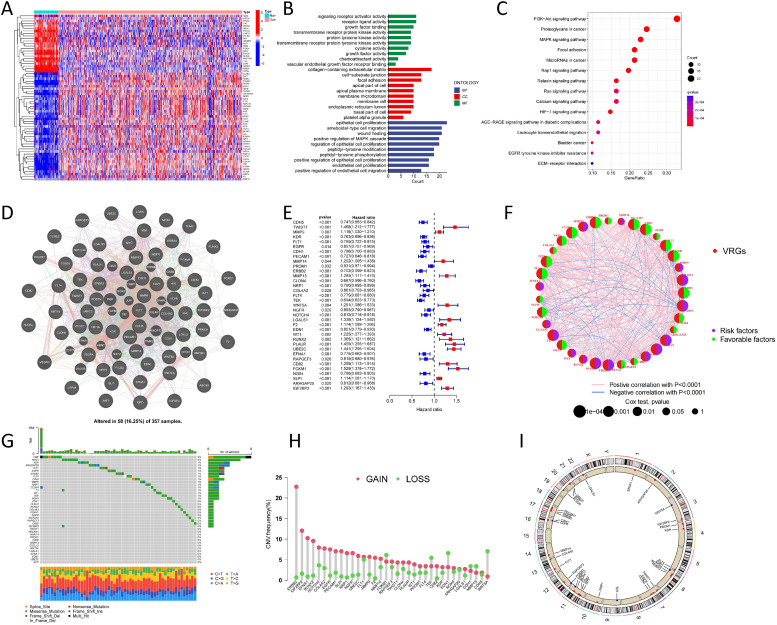
**Multi-omics Landscape of VRGs with Differential Expression in KIRC.** (A) Distribution of VRG expression in normal and KIRC. (B, C) Analysis of VRGs in KIRC using GO and KEGG. (D) PPI network among VRGs. The forest plot and circus plot depict the predictive value and associations of differentially expressed VRGs in KIRC patients (E, F). (G, H, I) Characteristics of VRGs in KIRC patients: mutation frequencies, CNVs, and chromosomal locations of these variants. (*p* < 0.05 *; *p* < 0.01 **; *p* < 0.001 ***).

The expression levels of 158 VRGs in tumor and normal specimens were determined utilizing the TCGA-KIRC dataset, yielding 44 upregulated genes and 22 downregulated genes (Fig. 1A), with |log2FC|>1. Subsequently, GO and KEGG analysis methods were employed to explore the functions, pathways, and potential associations with ccRCC occurrence of differential VRGs. GO analysis revealed the top five abundant GO_BP terms: "epithelial cell proliferation," "ameboid-type cell migration," "wound healing," "positive regulation of MAPK cascade," and "regulation of epithelial cell proliferation" (Fig. 1B). KEGG pathway analysis revealed that VRGs mainly participated in "PI3K-AKT signaling pathways", "MAPK signaling pathways", "HIF-1 signaling pathways", "Focal adhesion", and "ECM receptor interaction" (Fig. 1C). Protein-protein interaction (PPI) analysis unveiled strong interactions among VRGs (Fig. 1D). The comprehensive landscape of prognostic value and interactions of 35 differential VRGs was delineated through univariate Cox analysis and regulatory network depiction (Fig. 1E–F). Furthermore, the waterfall plot in Fig. 1G illustrated the mutational landscape of VRGs, with 18 mutations having a frequency >1 %, closely associated with ccRCC progression or recurrence. Extensive CNVs were detected in VRGs (Fig. 1H), with gene copy number amplification being the most common. Fig. 1I displays the locations of CNV changes on chromosomes. These findings suggest that CNVs may regulate the expression of VRGs. In conclusion, there are significant differences in genetic backgrounds and VRG expression levels between ccRCC and normal tissues, indicating the potential importance of VRGs in the initiation and progression of ccRCC.

### Generation of vascular mimicry subtypes in ccRCC

**Fig. 2:**
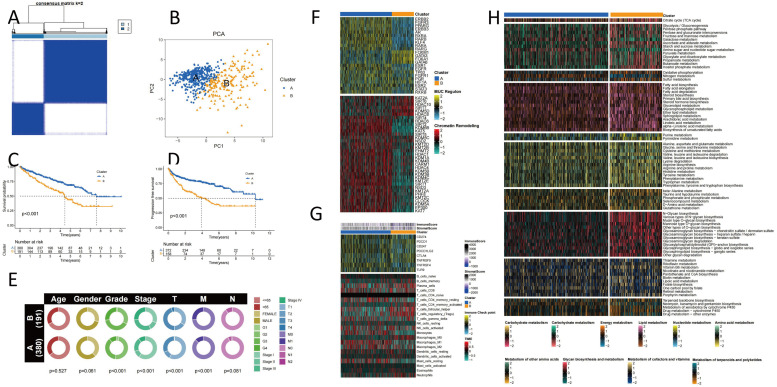
**VRGs Clusters and Their Biological Characteristics in KIRC.**(A) The consensus matrix heatmap illustrates the related areas and two clusters. (B)PCA reveals significant differences between the two subgroups. (C, D)Survival analyses for OS and PFS in TCGA-KIRC are presented for the two clusters. (E) The correlation of the two clusters with clinical characteristics is depicted. (F) The spectrum of regulators' regulatory activity for the two clusters. (G) Distribution of immune cells in two clusters. (H) ssGSEA enrichment analysis demonstrates variations in metabolic processes between the two clusters. (*p* < 0.05 *; *p* < 0.01 **; *p* < 0.001 ***).

An unsupervised clustering technique was employed to classify 571 cc RCC patients from TCGA-KIRC and GSE29609 based on the expression levels of 35 differentially expressed VRGs, aiming to elucidate the expression patterns of vascular mimicry. The analysis revealed that two clusters (*k* = 2) represented the optimal grouping (Fig. 2A). Patients from the entire cohort were distributed into Cluster A (*n* = 380) and Cluster B (*n* = 191). Various dimensionality reduction techniques were employed, including Principal Component Analysis (PCA) (Fig. 2B), 3D PCA (Supplementary Figure S1A), UMAP (Supplementary Figure S1B), and t-SNE (Supplementary Figure S1C), to validate the distinct separation of the two VRG clusters. Significant differences were observed in VRG expression between the two groups (Supplementary Figure S1D). Moreover, OS and PFS of the two clusters were discussed, revealing significant differences in survival rates (Fig. 2C, D). As shown in Fig. 2E, comparisons of clinical-pathological variables between the two clusters revealed significant differences in clinical characteristics. To further investigate transcriptional regulatory differences, potential regulatory factors associated with cancer chromatin remodeling and transcription factors playing significant roles in KIRC were analyzed (Fig. 2F). Regulators such as ERBB2, PPARG, FGFR3, and AR were significantly activated in Cluster A, while FOXM1, FOXA1, GATA3, and GATA6 were specifically enriched in Cluster B. Epigenetically driven transcriptional networks may play a significant role in distinguishing these molecular clusters, as indicated by the range of regulatory activities associated with carcinogenic chromatin remodeling. This further underscores the potential existence of distinct regulatory patterns between the clusters. Subsequently, the CIBERSORT method was employed to assess the differences in immune cell levels between the two clusters, as shown in Fig. 2G, revealing significant disparities in cells such as Tregs, Tfhs, and M1 macrophages between the two clusters.KEGG enrichment analysis was performed using GSVA, and the heatmap displayed (Supplementary Figure S1E) significant differences in several metabolic pathways between the two clusters. Subsequent quantification of metabolic pathways using ssGSEA revealed significantly increased metabolism, such as glycolipid metabolism, in Cluster B, while relatively lower in Cluster A (Fig. 2H). These results emphasize a strong correlation between ccRCC clusters defined based on VRGs, the prognosis of ccRCC patients, molecular characteristics, and metabolic status.

**Fig. 3:**
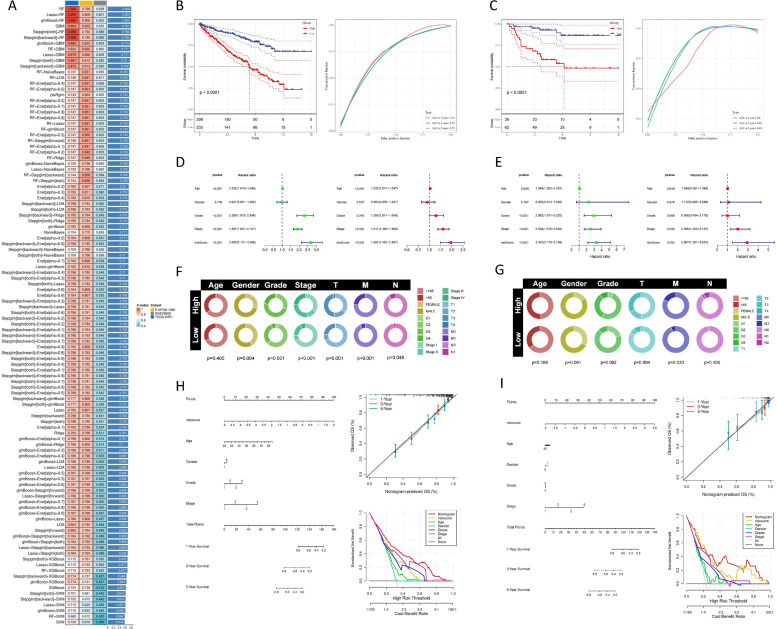
**Construction and Prognostic Value of VRG_Score.** (A) Rank combinations of machine learning algorithms based on their average AUC.(B, C) ROC curves and KM curves for TCGA-KIRC and E-MTAB-1980.(D, E) Univariate and multivariate analysis of clinical features and VRG_Score in the TCGA-KIRC and E-MTAB-1980.(F, G) Differences of clinical characteristics based VRG_Score in the TCGA-KIRC and E-MTAB-1980.(H, I)Nomogram created using clinical characteristics and VRG_Score from the TCGA-KIRC and E-MTAB-1980, along with calibration curves and DCA curves. (*p* < 0.05 *; *p* < 0.01 **; *p* < 0.001 ***).

### Development and validation of the prognostic VRG_Score

33 common VRGs were identified across the TCGA, E-MTAB-1980, and GSE29609 datasets, and then integrated into a unified framework to compute the VRG_score. In TCGA-KIRC, we established a consistent model based on combinations of 113 algorithms and calculated the average AUC value for each model across all cohorts to assess the predictive capability of all models. As depicted in Figure A, the RF algorithm maintained the highest average AUC value. Combining the RF algorithm with the Cox algorithm filtered out the most valuable model, which was constructed by 4 hub genes (Supplementary Table). The VRG_score was computed for each sample in the TCGA and E-MTAB-1980 cohorts, with AUCs for 1, 3, and 5-year OS of 0.74, 0.72, and 0.72 in TCGA-KIRC(Figs. 3B) and 0.8, 0.85, and 0.84 in E-MTAB-1980(Figs. 3C), indicating significantly shorter survival durations in the high-risk groups compared to the low-risk groups. Univariate and multivariate Cox regression analyses were conducted to assess independent prognostic variables associated with OS. The results demonstrated that the VRG_score was a significantly superior independent prognostic factor for ccRCC patients compared to other clinical features (Fig. 3D, E). Additionally, variations in clinical parameters were explored to further elucidate the role of VRG_score in the progression of ccRCC. High risk was associated with increased staging, grading, and TNM in the TCGA-KIRC cohort (Fig. 3F), and elevated grading, T, and M in the E-MTAB-1980 cohort (Fig. 3G). Finally, we developed a nomogram to predict OS using clinical parameters and VRG_score based on the two cohorts. Internal validation calibration plots of the nomogram demonstrated good consistency between predicted probabilities and actual observations of 1, 3, and 5-year OS. DCA curves revealed higher net benefits obtained with the nomogram in both TCGA and E-MTAB-1980 cohorts(Fig. 3H, I). The VRG_score also demonstrated outstanding predictive capability in an independent dataset, namely the ICGC-RECA-EU (Supplementary Figure S2). Collectively, these findings indicate that the VRG_score exhibits robust predictive performance, underscoring its applicability across diverse patient populations.

### Correlation between immune microenvironment features and VRG_Score

**Fig. 4:**
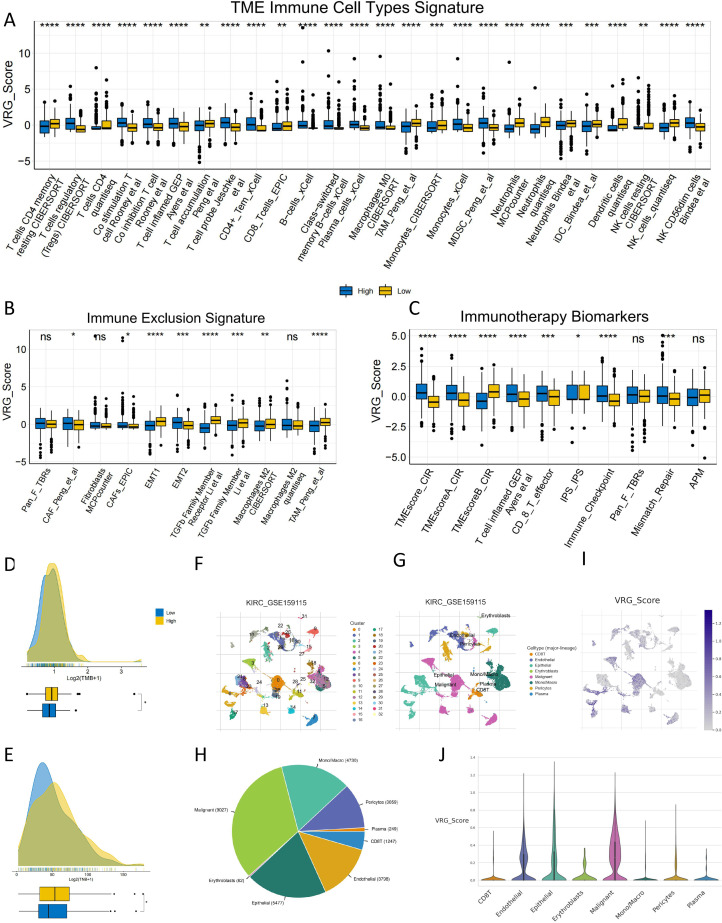
**Distribution of TME and Associated Molecular Features in Patients with Different VRG_Score.**(A-C) Distribution of immune cell types in the TME, immune exclusion features, AND immune therapy biomarkers between patients with different VRG_Score.(D, E) Distribution of TMB AND TNB between patients with different VRG_Score.(F-H) Annotation of all cell types and the percentage of each cell type in GSE159115.(I-J) Expression of VRG_Score in KIRC TME-associated cells.(*p* < 0.05 *; *p* < 0.01 **; *p* < 0.001 ***).

A comprehensive analysis of the TME in KIRC was conducted using ‘IOBR’ packages. Those with high VRG_Score exhibited higher levels of immune cell infiltration. This suggests that KIRC with high VRG_Score features an immune-activated state and is more likely to be classified as "hot tumors" (Fig. 4A). Given the significant enrichment of neutrophils and immune evasion-related molecular markers, patients with low VRG_Score tend to exhibit characteristics of "cold tumors" (Fig. 4B). As expected, the high VRG_Score group demonstrated a higher signature associated with immune therapy response (Fig. 4C). An evaluation of TMB and TNB was performed to assess the response to immune therapy in both groups of patients. The high VRG_Score group showed higher TMB and TNB, suggesting a potentially stronger immunogenicity in this group (Fig. 4D, E). Subsequently, further exploration was conducted into the correlation between VRG_score and the characteristics of immune checkpoint blockade (ICB) response, as well as the tumor immune cycle. As depicted in Supplementary Figure S3A, VRG_score exhibited a predominantly significant positive correlation with most features of ICB and steps of the tumor immune cycle. This further corroborated the effectiveness of VRG_score in predicting immune therapy response. Finally, we analyzed the expression of VRG_Score in the TME using the single-cell dataset GSE159115 from the TISCH2 database. In the GSE159115 dataset, there were 32 cell clusters and 8 cell types (Fig. 4F, G). Fig. 4H demonstrates the distribution and abundance of various cell types. VRG_Score was predominantly expressed in malignant tumor cells, endothelial cells, and epithelial cells(Fig. 4I, J).

### Drug sensitivity analysis in patients with different VRG_score

To identify potential therapeutic targets and compounds for patients with high VRG_score, we screened compounds highly correlated with VRG_score from three drug response databases (GDSC, CTRP, and PRISM). Firstly, we estimated the IC50 values of compounds from GDSC in TCGA-KIRC and performed Spearman correlation analysis with VRG_score. The four compounds with the largest negative correlation coefficients were Tozasertib, Schweinfurthin A, Teniposide, and Piperlongumine (Supplementary Figure S4A). Subsequently, we estimated the AUC values of compounds from CTRP and PRISM for each TCGA sample and conducted a correlation analysis between VRG_scores and estimated AUC values. For CTRP and PRISM, the scatterplots showed the four compounds with the largest negative correlation coefficients (CTRP: methotrexate, vincristine, leptomycin B, GSK461364; PRISM: gemcitabine, rubitecan, vincristine, cabazitaxel) (Supplementary Figure S4B, D), and compared their estimated AUC values among different VRG_score groups (Supplementary Figure S4C, E). Overall, all identified compounds showed significant negative correlations with VRG_score and may have useful therapeutic implications for high-risk ccRCC patients.

### Oncogenic role and prognostic value of upregulated FOXM1 in ccRCC

**Fig. 5:**
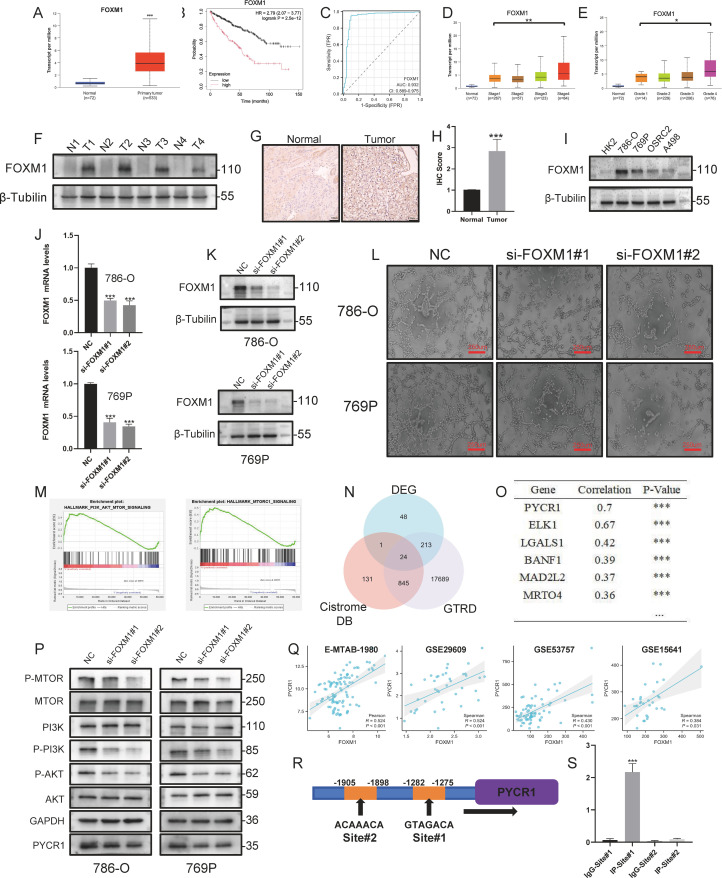
**FOXM1 Promotes ccRCC Progression and VM Formation while Upregulating PYCR1 Expression through Activation of the PI3K/AKT/mTOR Pathway.** (A)The UCLCAN database reveals differential expression of FOXM1,(B)KM curves, (C)ROC curves, and its distribution across (D)Stages and (E)Grades. (F)Western blot and (G, H)IHC depict the expression levels of FOXM1 in four pairs of tumors and adjacent tissues. (I)FOXM1 expression across different renal cancer cell lines. (J-K) RT-qPCR and Western blot experiments confirm the efficient knockdown efficiency of si-FOXM1. (L) After FOXM1 knockdown, the VM formation capability significantly decreases. (M) GSEA between different expression of FOXM1 in TCGA-KIRC. (N) Venn diagram depicting differentially expressed genes between different FOXM1 expression groups, intersected with potential FOXM1 target genes from GTRD and CistromeDB databases. (O) Correlation list of FOXM1 with intersecting genes in TCGA-KIRC.(P) Downregulation of FOXM1 markedly suppresses the expression of PYCR1 protein and activation of the PI3K/AKT/mTOR pathway. (Q) Correlation between FOXM1 and PYCR1 mRNA expression level in multiple datasets. (R)Predicted transcription binding motifs of FOXM1 and potential binding sites with PYCR1.(S) ChIP-PCR confirmed the binding of FOXM1 to the PYCR1 promoter. The scale bar of (H) is 120 µm, and the scale bar of (N) is 250 µm. (*p* < 0.05 *; *p* < 0.01 **; *p* < 0.001 ***).

To further confirm the role of VRGs in the pathogenesis of kidney cancer, an inquiry into the GEPIA database was conducted, revealing significant expression and survival differences of genes within the four VRG_Score models (Supplementary Figures 5A-D). As illustrated in Supplementary Figure 5E, the HPA database indicated expression variances of genes within the VRG_Score model in ccRCC. Similarly, qPCR experiments conducted on four pairs of ccRCC tissues and their corresponding adjacent non-tumor tissues also supported this observation (Supplementary Figures 5F-I). Given the pronounced expression difference of FOXM1 within the VRG_Score, and the limited reports linking FOXM1 to VM, FOXM1 was selected as the core gene of the VRG_Score to investigate its impact on VM in ccRCC.The UCLCAN database indicates that FOXM1 is highly expressed in renal cancer and is associated with deteriorated survival and disease progression in ccRCC patients, exhibiting excellent diagnostic performance(Fig. 5A, B, C, D, E). Western blot and IHC analyses demonstrate significantly higher expression of FOXM1 in ccRCC tissues compared to adjacent non-tumor tissues (Fig. 5F, G, H). Following comparison across multiple cell lines, we selected renal cancer cell lines 786-O and 769P to establish si-FOXM1 constructs (Fig. 5I). Western blot and rt-qPCR analyses confirm the robust knockdown efficiency of si-FOXM1#1 and #2 (Fig. 5J–K). Subsequent tube formation assays validate that downregulating FOXM1 significantly reduces the ability of 786-O and 769P to form VM (Fig. 5L). In conclusion, FOXM1 is likely to promote the VM of tumor cells, thereby accelerating the progression of KIRC.

### FOXM1 up-regulates PYCR1 expression and activates the PI3K/AKT/mTOR signaling pathway

An exploration into the downstream regulatory mechanisms through which FOXM1 promotes the malignant phenotype of ccRCC was undertaken.GSEA Analysis using the TCGA-KIRC revealed significant enrichment of the "PI3K/AKT/mTOR signaling pathway" and "MTORC1 signaling" pathways in patients with high FOXM1 expression (Fig. 5M). Protein imprinting analysis confirmed that knockdown of FOXM1 significantly reduced the phosphorylation of PI3 K, AKT, and mTOR (Fig. 5P). As for how FOXM1 activates the PI3K/AKT/mTOR pathway, it is hypothesized that FOXM1, as a transcription factor, may transactivate certain key genes. Therefore, a comparison was made between the transcriptome sequencing data of high and low FOXM1 expression from the TCGA dataset to identify differentially expressed genes, which were then cross-referenced with the GTRD [27] database and CistromeDB [28] predicted target genes, revealing 24 potential candidate genes (Fig. 5N). Among these, PYCR1 exhibited the strongest co-expression with FOXM1, piquing our interest (Fig. 5O). Data from other datasets (E-MTAB-1980, GSE29609, GSE53757 [29], and GSE15641 [30]) also displayed a strong correlation between PYCR1 and FOXM1 expression (Fig. 5Q). Previous studies have reported that PYCR1 can promote tumor proliferation by activating the PI3K/AKT/mTOR signaling pathway [31,32]. Following FOXM1 knockdown, PYCR1 showed simultaneous downregulation (Fig. 5P), strongly suggesting that FOXM1 is a transcription factor targeting PYCR1. The JASPAR database [33] also predicted potential binding sites between FOXM1 and PYCR1 promoter regions(Fig. 5R). Based on this, qPCR primers were designed for use in CHIP-qPCR experiments. Indeed, amplification of target products was observed through immunoprecipitation with FOXM1 antibodies(Fig. 5S). These results collectively demonstrate that FOXM1, as a transcriptional activator, positively regulates PYCR1 expression.

**Fig. 6:**
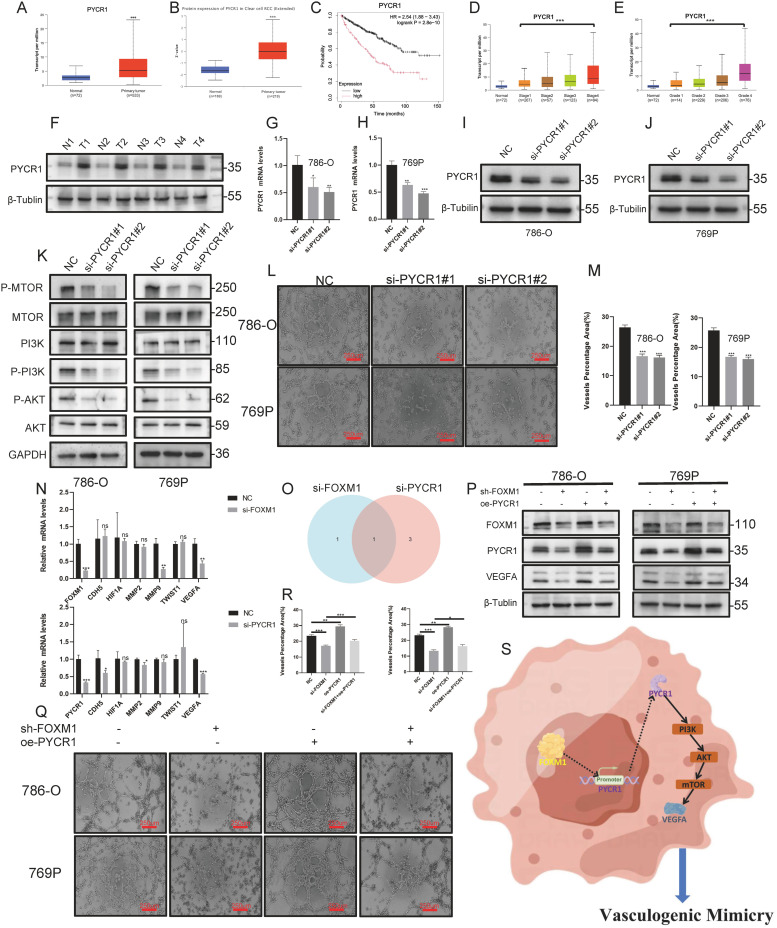
**PYCR1 Mediates VM Formation in ccRCC through FOXM1 and PI3K/AKT/mTOR Signaling, with VEGFA as a Critical Regulator** The UCLCAN database was utilized to analyze the differences in mRNA(A) and protein (B) expression of PYCR1 between tumor and normal tissues. KM curves (C), as well as the distribution across staging and grading (D, E), were examined. (F) Expression levels of PYCR1 in tumors and corresponding normal tissues. (G-J)The effectiveness of si-PYCR1 knockdown was confirmed by RT-qPCR and Western blotting. (K)Downregulation of PYCR1 markedly suppresses activation of the PI3K/AKT/mTOR pathway. (L-M)Following the deletion of PYCR1, there was a significant decrease in VM formation ability. (N) Changes in VM gene expression after FOXM1 and PYCR1 knockout. (O) Venn diagram showing the intersection of target genes for both. PYCR1 overexpression partially rescues weakened VEGFA downregulation(P) and VM formation (Q, R) caused by FOXM1 knockout. (S) Schematic diagram of the mechanism in this study. The scale bar of (L, Q) is 250 µm. (*p* < 0.05 *; *p* < 0.01 **; *p* < 0.001 ***).

To further elucidate the role of PYCR1 in promoting VM in ccRCC, we utilized the UCLCAN database and observed a significant upregulation of PYCR1 at both mRNA and protein levels in ccRCC(Fig. 6A, B). Additionally, patients with elevated PYCR1 exhibited poorer prognosis, staging, and grading(Fig. 6C–E). Western blot was conducted to explore the expression levels of PYCR1 in four pairs of tumors and corresponding normal tissues(Fig. 6F). Subsequently, PYCR1 was knocked down in 786-O and 769P cell lines, and this was validated using protein immunoblotting and RT-qPCR(Fig. 6G–J). Following these experiments, we found that PYCR1 knockdown significantly inhibited the levels of the PI3K/AKT/mTOR pathway and the formation of VM in ccRCC(Fig. 6K–M).

### FOXM1 activates VEGFA regulation and ccRCC VM through the PYCR1/PI3K/AKT/mTOR axis

As depicted in Fig. 6N, two genes were significantly downregulated after si-FOXM1 treatment, and four genes after si-PYCR1 treatment, as assessed by qPCR. We identified VEGFA as a downstream target of the FOXM1/PYCR1/PI3K/AKT/mTOR axis through gene intersection (Fig. 6O). Western blotting showed si-FOXM1 reduced VEGFA levels, while oe-PYCR1 increased them, and could rescue VEGFA reduction from FOXM1 knockdown (Fig. 6P). PYCR1 overexpression also rescued VM decline due to FOXM1 knockdown (Fig. 6Q, R). This indicates the FOXM1/PYCR1/PI3K/AKT/mTOR/VEGFA pathway promotes ccRCC VM formation. The study's mechanism is summarized in Fig. 6S.

## Discussion

ccRCC being the predominant pathological subtype of RCC, stands as one of the most prevalent malignancies globally, contributing significantly to cancer-related mortality [1]. Despite surgical resection combined with systemic chemotherapy being considered the primary treatment strategy for ccRCC, patients undergoing surgical resection still face the risk of recurrence and metastasis [34]. For patients with locally advanced, recurrent, or metastatic ccRCC who are not amenable to surgical resection, targeted therapy, and immunotherapy have become standard choices for systemic treatment [35]. However, the clinical efficacy of anti-angiogenic therapy is not satisfactory [36]. Therefore, it is imperative to search for new factors to predict the prognosis of ccRCC patients.

VM, an alternative blood supply independent of endothelial cells, has been demonstrated as a critical factor in tumor progression, closely associated with increased chemoresistance, lower survival rates, and poor prognosis in cancer patients [37]. Furthermore, the VM structure may serve as a scaffold to support the recruitment of mononuclear cells, indicating a potential connection between the VM and the TME [38]. In this study, we retrieved VRGs from the Genecards database and integrated TCGA-KIRC data to identify prognostically significant differential VRGs. Subsequently, consensus clustering identified two prognostic subtypes with distinct features, exhibiting significant differences in chromatin remodeling, metabolic pathways, and clinical characteristics between the two subgroups. Overcoming the limitations imposed by algorithm selection, we constructed the VRG_score signal using 113 algorithm combinations, demonstrating a more robust predictive performance compared to previous studies focusing on VM. This contrasts sharply with the studies by Liang et al. [39] and Yang et al. [40], which utilized only a single linear regression model and displayed suboptimal performance in external validation. The VRG_score signal exhibited superior predictive performance compared to other conventional clinical indicators, as evidenced by survival curves, ROC curves, nomograms, and calibration curves. Additionally, there was good agreement between predicted and observed values, providing theoretical support for clinical decision-making.

As a member of the FOX protein family, forkhead box M1 (FOXM1) is deemed essential in cancer progression, regulating important aspects such as cancer cell growth, metastasis, recurrence, and stem cell characteristics [41]. Recent studies have highlighted FOXM1′s involvement in various cancers, including bladder cancer [42], prostate cancer [43], glioblastoma [44], and breast cancer [45]. As a representative transcription factor of the FOX protein family, FOXM1 has been extensively studied for its significant role in pan-cancer, making it a focal point of our research. We systematically analyzed the FOXM1 gene in ccRCC and found elevated expression levels of FOXM1, which were indicative of poor prognosis in ccRCC. Through experiments, we validated FOXM1′s promotion of VM formation in ccRCC. Its downstream regulation of PYCR1 through positive transcriptional control activates the PI3K/AKT/mTOR signaling pathway. This implies that FOXM1, as an oncogenic regulatory factor, can strongly regulate VM formation and may serve as a new target for ccRCC therapy.

Despite the utility of VRG_score in the therapeutic context of ccRCC, this study has several limitations. First, the study relied on retrospectively generated datasets, which may introduce selection and information biases, thereby limiting causal inferences. This necessitates further validation of the VRG_score in prospectively designed cohorts that include comprehensive clinicopathological characteristics and survival data. Furthermore, a comprehensive exploration of the biological implications of the genes included in VRG_score has not yet been carried out; this gap highlights the need for further mechanistic research. The functions of certain genes still require further investigation, indicating a need to deepen our understanding of their roles in ccRCC. Moreover, despite the good predictive ability of VRG_score, its potential application in clinical practice requires further investigation, particularly regarding its integration with existing clinical scoring systems.

Based on the results of our study, future research should prioritize the following directions: Firstly, further exploration of the functions and mechanisms of VRGs in the progression of ccRCC is needed, particularly their specific roles in the TME and their interactions with immune cells. Secondly, future interdisciplinary studies should integrate genomics, epigenetics, and tumor metabolism to achieve a comprehensive understanding of FOXM1 and other VRGs in ccRCC. Finally, in terms of the clinical application of VRG_score, further research should investigate its adaptability across various treatment modalities, particularly in the combined use of targeted therapy and immunotherapy.

## Conclusion

This study identified two molecular clusters of ccRCC through consensus clustering of VRGs and revealed significant differences in prognosis, immune response, and metabolism, thereby providing important references for clinicians in making personalized treatment decisions. Additionally, leveraging a machine learning algorithm framework, the VRG_Score was defined, and the TME characteristics of VRGs were explored along with their efficacy as biomarkers for treatment response, demonstrating their potential utility in clinical applications. Finally, we elucidated the crucial role of the FOXM1-PYCR1-PI3K/AKT/MTOR-VEGFA axis in regulating VM formation in ccRCC, which not only enhances the understanding of the biological mechanisms underlying VM formation in ccRCC but also provides novel insights into the molecular mechanisms and therapeutic targets for the development of renal cell carcinoma.

## List of abbreviations

Renal cell carcinoma (RCC)

Clear cell renal cell carcinoma (ccRCC)

Vasculogenic mimicry (VM)

Vascular Mimicry-Associated Genes (VRGs)

Fragments Per Kilobase of exon model per Million mapped fragments (FPKM)

Transcripts per million (TPM)

Kidney Renal Clear Cell Carcinoma (KIRC)

Tyrosine kinase inhibitors (TKIs)

The Cancer Genome Atlas (TCGA)

Copy Number Variation (CNV)

Geostationary Orbit(GEO)

International Cancer Genome Consortium(ICGC)

Gene Ontology (GO)

Kyoto Encyclopedia of Genes and Genomes (KEGG)

Protein-protein interaction (PPI)

Kaplan-Meier (KM)

Overall Survival (OS) progression-free survival (PFS)

Gene Ontology Biological Process (GO_BP),

Gene Ontology Cellular Component (GO_CC),

Gene Ontology Molecular Function (GO_MF)

Gene set variation analysis (GSVA)

Single-sample gene set enrichment analysis (ssGSEA)

Gene Set Enrichment Analysis (GSEA)

Principal Components Analysis(PCA)

Uniform Manifold Approximation and Projection (UMAP)

T-Distributed Stochastic Neighbor Embedding (t-SNE)

Least absolute shrinkage and selection operator (Lasso)

Stepwise Generalized Linear Model (Stepglm)

Extreme gradient boosting machine (XGBoost)

Random Forest (RF)

Elastic Net (Enet)

Partial Least Squares Regression for Generalized Linear Models (plsRglm)

Generalized Boosted Regression Modeling (GBM)

Linear Discriminant Analysis (LDA)

Generalized Linear Model Boosting (glmBoost)

Support Vector Machine (SVM)

Receiver operating characteristic (ROC)

Area under the receiver operating characteristic curve (AUC)

Decision curve analysis (DCA)

Tumor mutational burden (TMB)

Tumor neoantigen (TNB)

Tumor microenvironment (TME)

Half maximal inhibitory concentration (IC50)

Short tandem repeat (STR)

Fetal bovine serum (FBS)

Quantitative Fluorescence Polymerase Chain Reaction (RT-qPCR)

Immunohistochemistry (IHC)

Chromatin Immunoprecipitation (CHIP)

Immune checkpoint blockade (ICB)

## Ethics approval and consent to participate

The studies involving human participants were reviewed and approved by the Ethics Committee of the Second Hospital of Hebei Medical University (2024-R205).

## Data availability

All data are available in a public, open-access repository. The data that support the findings of this study are available from the corresponding author upon reasonable request.

## Consent for publication

All authors have read and approved the final version of the manuscript for publication.

## Funding

This study received partial support from the 10.13039/501100001809National Natural Science Foundation of China (No. 82072842), the Natural Science Foundation of Hebei Province (No. H2022206199), and the Graduate Innovation Funding Project of Hebei Medical University (XCXZZS202319).

## CRediT authorship contribution statement

**Chao Xu:** Methodology, Formal analysis, Conceptualization. **Sujing Zhang:** Methodology, Formal analysis, Conceptualization. **Jingwei Lv:** Software. **Yilong Cao:** Software. **Yao Chen:** Visualization. **Hao Sun:** Visualization. **Shengtao Dai:** Writing – review & editing. **Bowei Zhang:** Writing – review & editing. **Meng Zhu:** Validation. **Yuepeng Liu:** Validation. **Junfei Gu:** Supervision, Project administration.

## Declaration of competing interest

The authors declare no competing interests.

## Appendix Supplementary materials

Image, application 1

Image, application 2

Image, image 3

Image, image 4

Image, image 5

Image, image 6

Image, image 7
