# Supplementary material for: Machine learning identification of a novel vasculogenic mimicry-related signature and FOXM1’s role in promoting vasculogenic mimicry in clear cell renal cell carcinoma
Source: Transl Oncol. 2025 Feb 3;53:102312. doi: 10.1016/j.tranon.2025.102312 (PMC11847097; doi:10.1016/j.tranon.2025.102312)
Supplement: Supplementary file 2 [file mmc2.doc]

**Supplementary Figure S1: Identification of gene subgroups based on DEGs.** 3D PCA (A), UMAP (B), and t-SNE (C) were utilized to assess the separation of VRG clusters. (D) Differential expression of VRGs between two gene clusters, and (E) GSVA of biological pathways between the two gene clusters. (p < 0.05 *; p < 0.01 **; p < 0.001 ***).

**Supplementary Figure S2: Prognostic Value of VRG_Score in ICGC-RECA-EU.** (A, B)Kaplan-Meier (KM) survival curves and Receiver Operating Characteristic (ROC) curves for the ICGC-RECA-EU cohort. (C, D) Univariate and multivariate analyses of clinical features and VRG_Score within the TCGA-KIRC and ICGC-RECA-EU datasets. (E) Differences in clinical characteristics based on VRG_Score in the ICGC-RECA-EU cohort. (F, G, H) The nomogram was developed utilizing clinical characteristics and VRG_Score from the ICGC-RECA-EU, accompanied by calibration curves and Decision Curve Analysis (DCA) curves. (*p < 0.05; **p < 0.01; ***p < 0.001)

**Supplementary Figure S3: Prediction of immune therapy responsiveness by VRG_score.** (A) Correlation of risk scores with ICB response features and each step of the tumor immune cycle.

**Supplementary Figure S4: Screening of potentially applicable drugs based on VRG_score.** (A) Spearman correlation analysis of AMrs and estimated IC50 values in the GDSC database. The four compounds with the largest negative correlation coefficients are Tozasertib, Schweinfurthin A, Teniposide, and Piperlongumine. (B & D) Estimation of AUC values from compounds from CTRP and PRISM for each TCGA sample, and Spearman correlation analysis of AMrs and estimated AUC values. For CTRP and PRISM, the scatterplots show the four compounds with the largest negative correlation coefficients (CTRP: methotrexate, vincristine, leptomycin B, GSK461364; PRISM: gemcitabine, rubitecan, vincristine, cabazitaxel), and (C & E) all estimated AUC values of these compounds are significantly lower. (p < 0.05 *; p < 0.01 **; p < 0.001 ***).

**Supplementary Figure S5: illustrates the expression and survival differences of VRG_Score component genes (FOXM1, ERBB2, TEK, CLDN4) in ccRCC.** Panels (A-D) depict the expression levels of VRG_Score component genes in ccRCC, along with overall survival (OS) and disease-free survival (DFS) differences, as analyzed through GEPIA. Panel (E) illustrates the expression differences of VRG_Score component genes in ccRCC using data from the Human Protein Atlas (HPA) database. Panels (F-I) show the qPCR validation of the expression of VRG_Score component genes in four pairs of ccRCC and adjacent non-tumor tissues.(p < 0.05 *; p < 0.01 **; p < 0.001 ***).
