# Supplementary figures and images for: Machine learning identification of a novel vasculogenic mimicry-related signature and FOXM1’s role in promoting vasculogenic mimicry in clear cell renal cell carcinoma
Source: Transl Oncol. 2025 Feb 3;53:102312. doi: 10.1016/j.tranon.2025.102312 (PMC11847097; doi:10.1016/j.tranon.2025.102312)

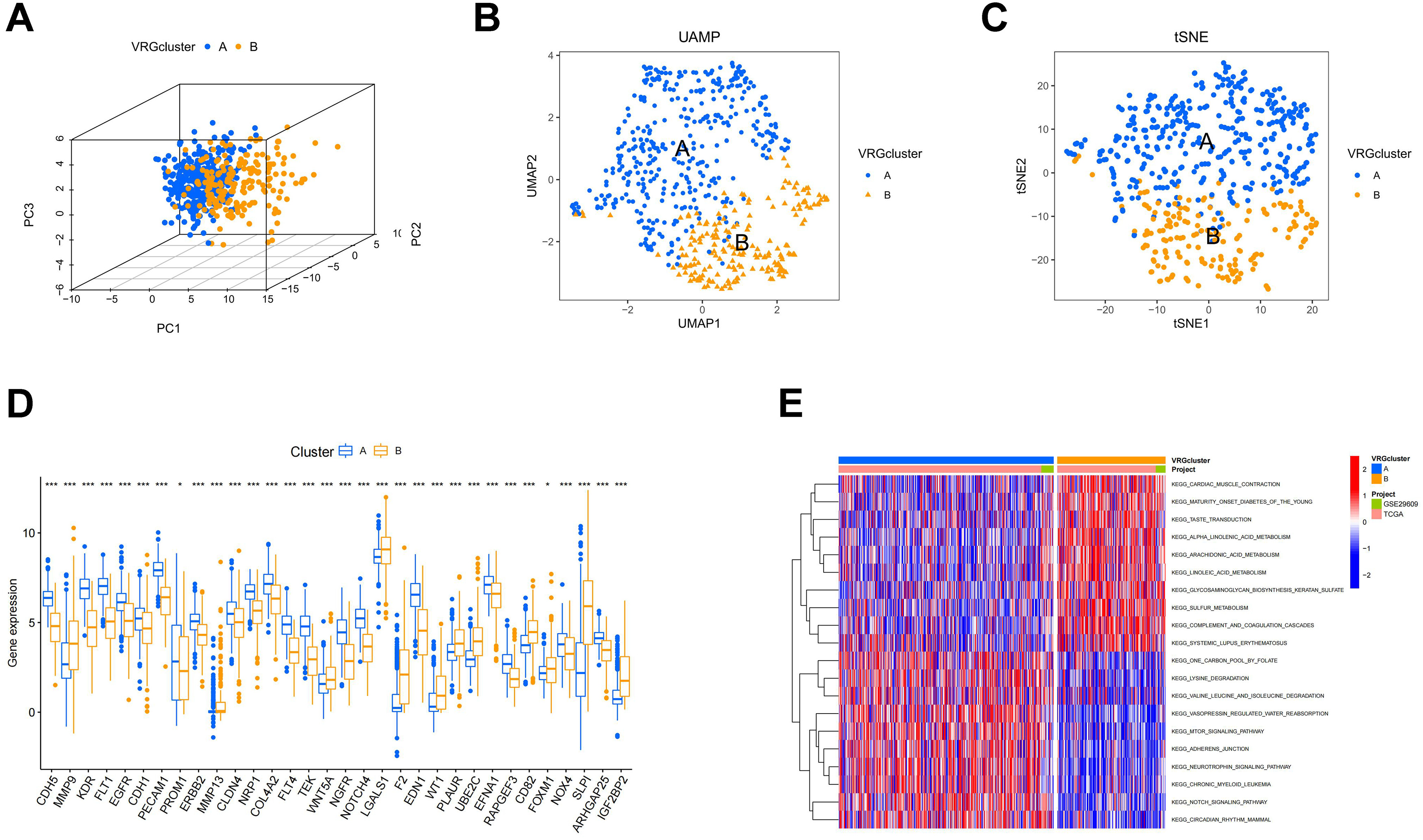

Supplement: Supplementary file 3 [file mmc3.jpg]

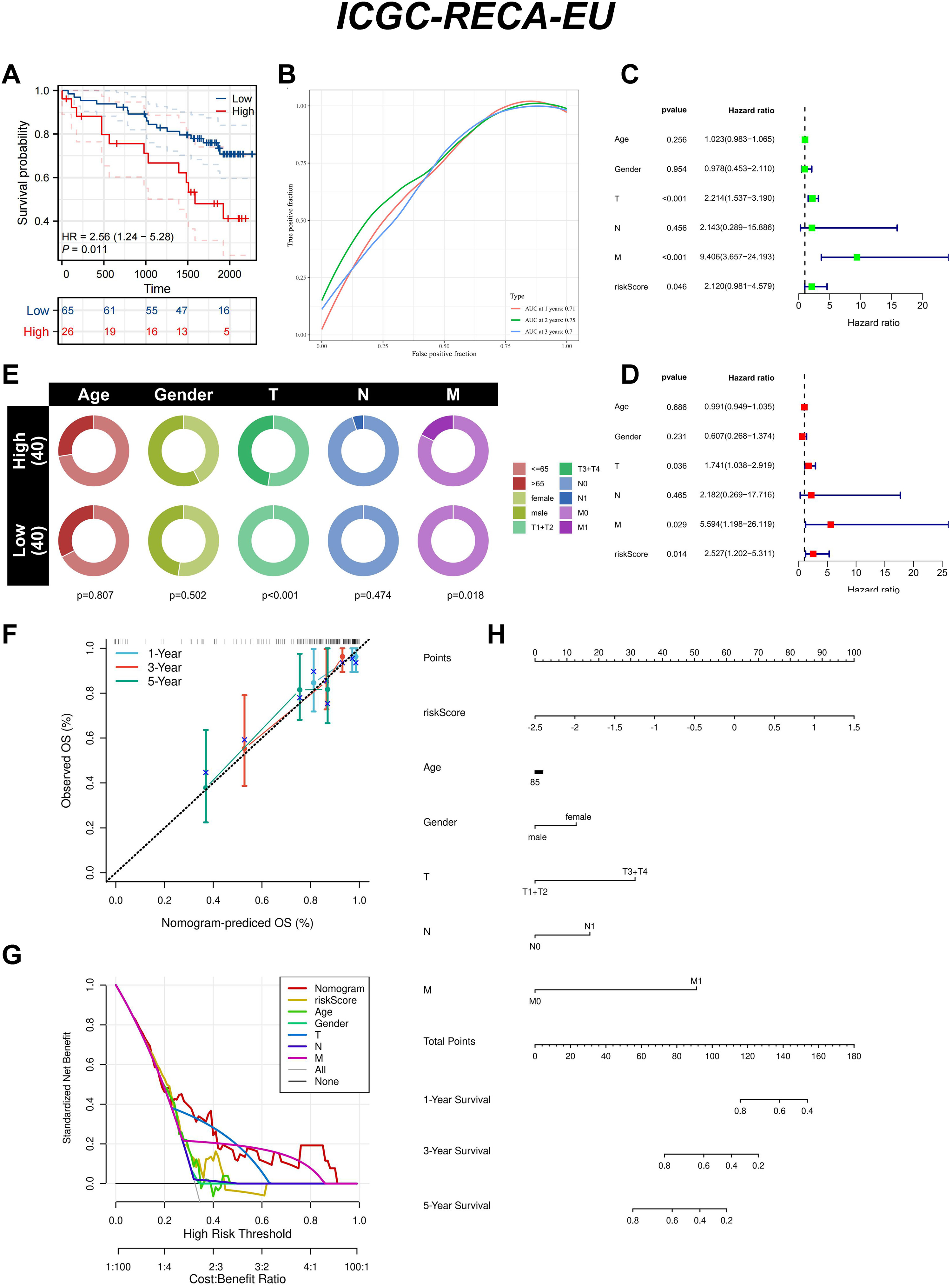

Supplement: Supplementary file 4 [file mmc4.jpg]

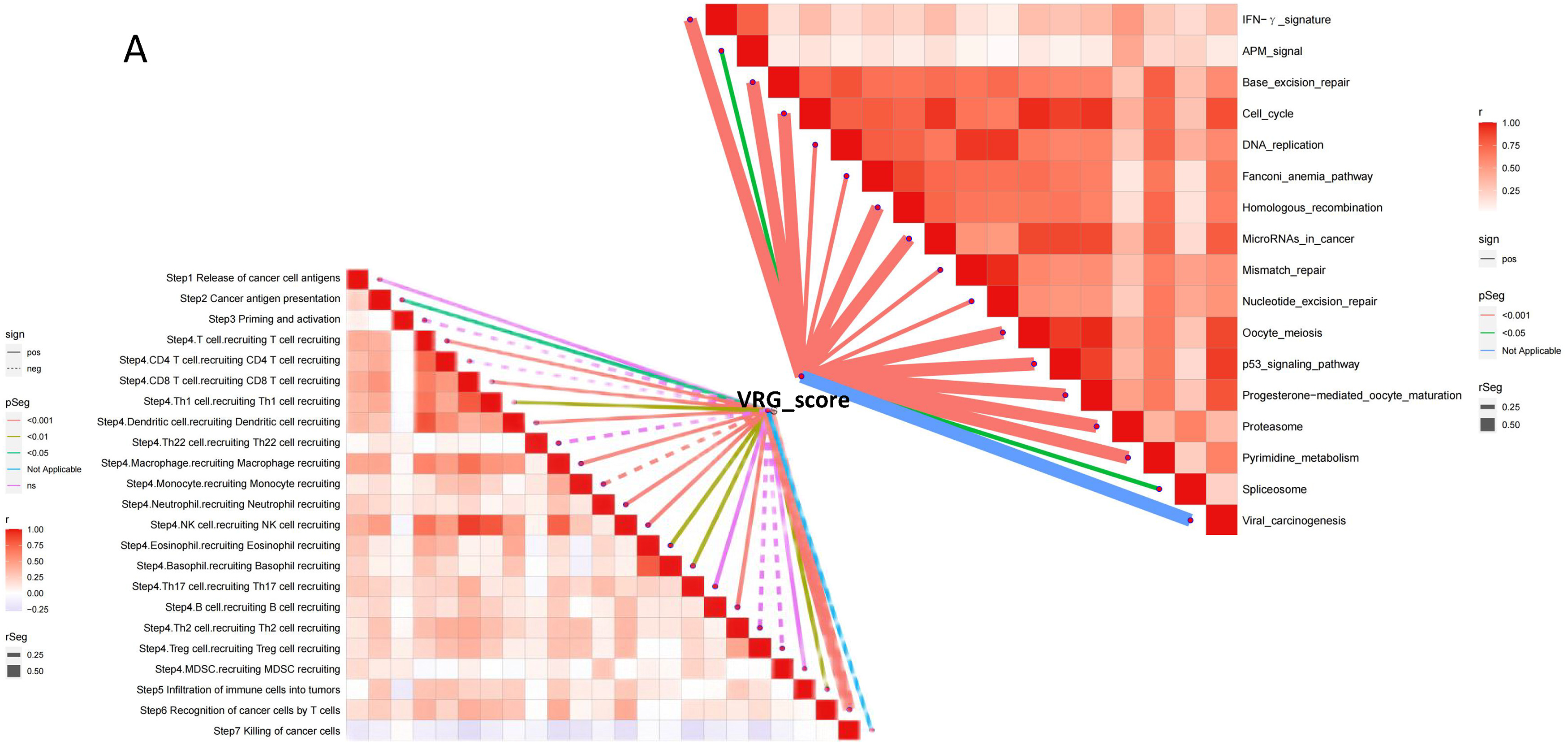

Supplement: Supplementary file 5 [file mmc5.jpg]

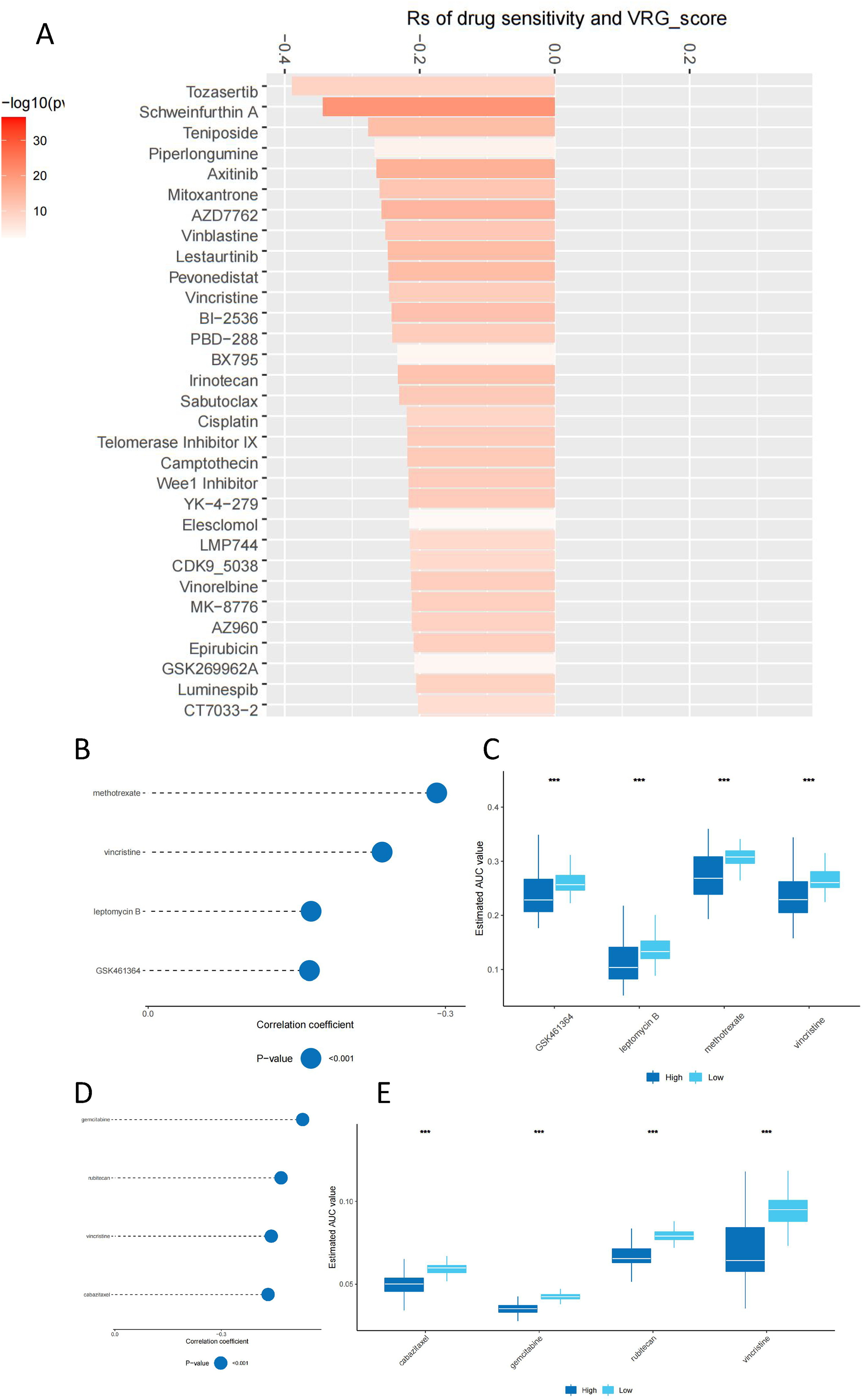

Supplement: Supplementary file 6 [file mmc6.jpg]

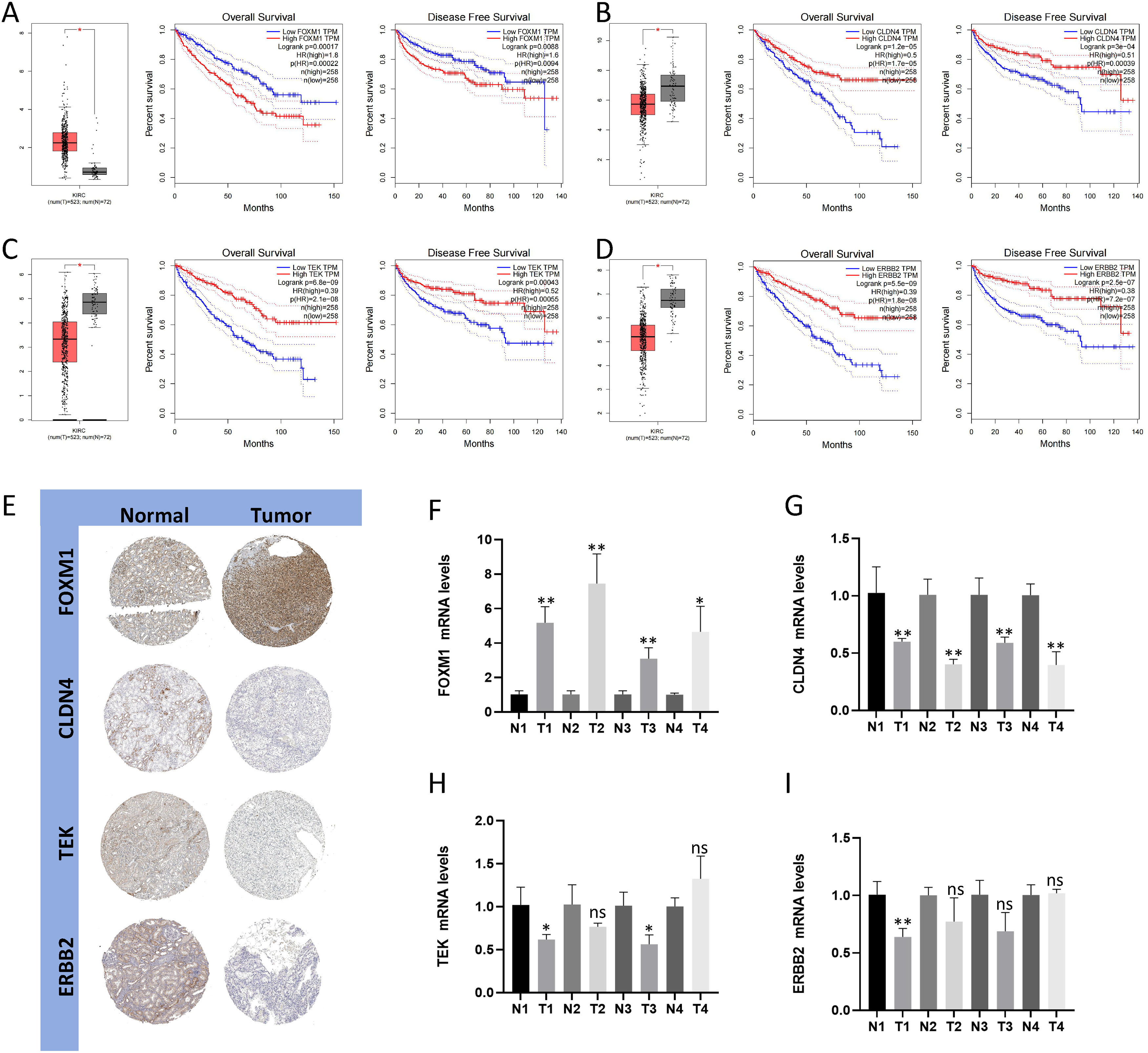

Supplement: Supplementary file 7 [file mmc7.jpg]
